# Modification of NO-cGMP Pathway Differentially Affects Diazepam- and Flunitrazepam-Induced Spatial and Recognition Memory Impairments in Rodents

**DOI:** 10.1007/s12640-019-00110-1

**Published:** 2019-12-02

**Authors:** Jolanta Orzelska-Górka, Piotr Bernat, Piotr Tutka, Joanna Listos, Ewa Kędzierska, Sylwia Fidecka, Sylwia Talarek

**Affiliations:** 1 Chair and Department of Pharmacology and Pharmacodynamics, Medical University of Lublin, Chodzki 4A, 20-093 Lublin, Poland; 2 Department of Experimental and Clinical Pharmacology, University of Rzeszów, Al. Kopisto 2a, 35-959 Rzeszów, PL Poland; 3 Laboratory for Innovative Research in Pharmacology, University of Rzeszów, Warzywna 1a, 35-959 Rzeszów, PL Poland; 4 National Drug and Alcohol Research Centre, University of New South Wales, Sydney, NSW Australia

**Keywords:** Novel object recognition, Spatial memory, Benzodiazepines, Nitric oxide, Mice/rats

## Abstract

This study investigated the influence of sildenafil and methylene blue (MB), two modulators of the nitric oxide (NO)-cyclic guanosine-3′,5′-monophosphate (cGMP) pathway on amnesic effects of two benzodiazepines (BZs) (diazepam (DZ) and flunitrazepam (FNZ)), in rodents—mice and rats. In the modified elevated plus maze (mEPM) and novel object recognition (NOR) tests, MB given ip at a dose of 5 mg/kg 5 min prior to DZ administration (0.25 or 1 mg/kg, sc) enhanced/induced memory impairment caused by DZ. When MB (2.5, 5, and 10 mg/kg) was applied 5 min prior to FNZ administration (0.05 and 0.1 mg/kg), an effect was opposite and memory impairment induced by FNZ was reduced. When sildenafil (2.5 and 5 mg/kg, ip) was applied 5 min prior to DZ, we observed a reduction of DZ-induced memory deficiency in the mEPM test. A similar effect of sildenafil was shown in the NOR test when the drug was applied at doses of 1.25, 2.5, and 5 mg/kg prior to DZ. In the mEPM test, sildenafil at abovementioned doses had no effects on FNZ-induced memory impairment. In turns, sildenafil administered at doses of 2.5 and 5 mg/kg increased the effect of FNZ on memory impairment in the NOR test. In conclusion, the NO-cGMP pathway is involved differentially into BZs-induced spatial and recognition memory impairments assessed using the NOR and mEPM tests. Modulators of the NO-cGMP pathway affect animal behavior in these tests in a different way depending on what benzodiazepine is applied.

## Introduction

Benzodiazepines (BZs) are one of the most commonly prescribed anxiolytic drugs. Only in the last decade, European Union Early Warning System, part of European Monitoring Centre for Drugs and Drug Addiction (EMCDDA), recognized 23 new psychoactive registered substances that fall into a BZs category (EMCDDA 2018). An access to BZs is relatively easy through medical health professionals but as well from illegal market. A call for precaution is needed, especially among a high-risk population where usage of couple neuroactive substances is applicable. BZs can cause a lot of side reactions, e. g., drowsiness, confusion, dizziness, trembling, impaired coordination, and disturbances of memory. Among many adverse effects, impairment of memory performance is an adverse effect that limits BZs use in such conditions as anxiety, insomnia, and seizures (EMCDDA 2018).

Memory has been a focal point of interest among scientists for centuries (McGaugh 2000). We can distinguish three types of memory classes and one of them, short-term memory, seems to be the most affected by BZs (Griffin et al. 2013). These drugs impair the episodic memory that enables to recall personally experienced events. Memory is composed of three sequential stages: acquisition, consolidation, and retrieval (McGaugh 2000). BZs induce the anterograde amnesia caused by their negative effects on the first stage of the memory process, i.e., acquisition. Through this mechanism, BZs affect a type of learning that depends on building new associations in memory and impairing acquisition of novel information (Griffin et al. 2013).

An amnesic effect of BZs is mediated by the activation of the γ-aminobutyric acid (GABA)_A_ receptors in the central nervous system (CNS). The GABA_A_ receptors are chloride-selective ion channels which are composed of different subunits: 2α, 2β, and 1γ. BZs have one binding site on each receptor GABA_A_ complex in a specific pocket created by the α and γ subunits. As a result of this binding, the chloride channel is opened and the chloride ions can pass through it, which causes hyperpolarization of the cell membrane. Subsequently, BZs enhance GABA-mediated neuronal inhibition (Buffett-Jerrott and Stewart 2002). Additionally, it has been reported that amnesic action of BZs is also caused by the modifications in hippocampal synaptic transmission along with plastic changes on cell membranes. In a specific region of the hippocampus (CA1), there are the BZs binding sites on GABA_A_ receptors. Moreover, it is regarded that BZs interference with long-term potentiation (LTP), which is known to be an important mechanism, contributes to learning and memory processes (Griffin et al. 2013).

Nitric oxide (NO) is a unique bioactive molecule that plays a vital role in a wide range of physiological and pathophysiological processes. l-Arginine is a substrate for NO formation. A family of NO synthases (NOS) catalyzes NO synthesis. We can distinguish four genetically different types of NOS: endothelial (eNOS), neuronal (nNOS), inducible (iNOS), and mitochondrial (mtNOS). It has been demonstrated that NO acts as a second messenger and/or a neurotransmitter. Unlike typical neurotransmitters, NO does not bind to receptors on neural membranes but it interacts with a specific target for NO—soluble guanylyl cyclase (sGC). Binding of NO to the heme group of sGC significantly increases activity of this enzyme to produce the second messenger of cyclic guanosine-3′, 5′-monophosphate (cGMP). cGMP plays an important function in NO signaling and in the regulation of physiologic responses. The phosphodiesterase type 5 (PDE5) degrades cGMP which leads to a decrease of the NO effect. This biochemical reaction is a target for sildenafil, a potent and selective inhibitor of PDE5 (Friebe and Koesling 2003; Polakowska et al. 2016).

It is considered that NO plays an important role in LTP, through which it affects learning and memory processes. It has been found that the inhibition of NOS activity impairs cognitive reactions in different rodents’ models of memory (e.g., modified elevated plus maze (mEPM), T-maze, Y-maze, the step-down passive avoidance, and novel object recognition (NOR)). NO donors, such as l-arginine and molsidomine, can reverse these detrimental effects (for review, see Prast and Philippu 2001; Pitsikas 2015). In addition, it has been observed that deficiency in learning and memory processes is associated with some pathological conditions, for example, epilepsy and stress, which may also result from mechanisms related to changes in the NO activity in the brain. NO donors, such as sodium nitro-prusside (SNP) or molsidomine, may be effective in the prevention of cognitive impairment caused by those conditions (Vanaja and Ekambaram 2011).

NO has the ability to evoke the release and retrograde uptake of several neurotransmitters in the brain, including GABA (Kuriyama and Ohkuma 1995; Tutka et al. 2007). For example, an increase in NO concentration in the brain is associated with the release of GABA in the cerebral cortex, hippocampus, and striatum (Segovia and Mora 1998). It may also play a significant role in the modulation of hippocampal GABAergic transmission (Szabadits et al. 2011; Vincent 2010). Our previous studies demonstrated that NO can enhance the antinociceptive (Talarek and Fidecka 2002), hypnotic (Talarek and Fidecka 2004), or anticonvulsant (Talarek and Fidecka 2003) effects of BZs in mice. Moreover, NO may play the role in the development of tolerance to some effects of BZs, such as sedation and coordination disturbance (Talarek et al. 2008, 2010).

In the previous studies, we demonstrated that single sc administration of BZs could affect memory processes in mice. Diazepam (DZ) and flunitrazepam (FNZ) impaired acquisition in the mEPM and the NOR tests. Interestingly, these studies revealed differences in the action of NOS inhibitors on memory impairment induced by DZ or FNZ (Orzelska et al. 2013, 2015; Orzelska-Gorka et al. 2016). The main aim of this research was to determine whether sildenafil, an inhibitor of PDE5, and methylene blue (MB), an inhibitor of NO-sensitive sGC, affect amnesic effects caused by DZ and FNZ in the mEPM and NOR tests in mice.

## Materials and Methods

### Animals

Experiments were carried out on 2-month-old male albino Wistar rats (Farm of Laboratory Animals, Z. Lipiec, Brwinow, Poland), weighing 200–250 g, and male albino Swiss mice (Farm of Laboratory Animals, Warsaw, Poland), weighing 20–25 g. After 1 week of adaptation to laboratory conditions, the animals were randomly assigned to experimental groups consisting of 10 mice or rats per group. The mice were maintained on a standard light-dark cycle and ambient temperature 18–22 °C with free access to chow pellets (Agropol, Motycz, Poland) and water. The experiments were performed between 9:00 and 17:00 h. All behavioral experiments were carried out according to the European Community Council Directive for Care and Use of Laboratory Animals (2010/63/EU) and approved by the Local Ethics Committee (37/2010).

### Drugs

Sildenafil, MB, and FNZ were purchased from Sigma Chemicals (St. Louis, USA). FNZ was dissolved in 0.5% Tween-80 (Sigma-Aldrich, St. Louis, MO, USA) (1–2 drops), gently warmed, and diluted with sterile saline solution (0.9% NaCl). DZ (Relanium, Polfa, Poland), sildenafil, and MB were diluted in 0.9% saline. All drug suspensions/solutions were prepared immediately prior to injection. Sildenafil and MB were given intraperitoneally (ip), whereas DZ and FNZ subcutaneously (sc). All drugs were injected in a volume of 0.2 ml per 100 g body weight (for rats) or in a volume of 0.1 ml per 10 g body weight (for mice). Control animals were administered a corresponding vehicle.

### Modified Elevated Plus Maze Test (mEPM)

A plus maze was made of dark plexiglass and consisted of two open arms (50 × 10 cm) and two enclosed arms (50 × 10 × 40 cm) arranged such that two open arms were opposite each other. The arms were connected by a central platform (10 × 10 cm). The apparatus was shaped like a “plus” sign and was elevated to a height of 50 cm above the floor (Itoh et al. 1990; Hlinak and Krejci 2002; Orzelska et al. 2013). The plus maze was placed in a dark room illuminated only by a halogen lamp oriented toward the central platform and giving a uniform dim light in the apparatus (intensity of 10 lx).

While an acquisition session (on day 1), each mouse was gently placed at the distal end of an open arm of the apparatus facing the central platform. The time it took mice to move from the open arm to one with the enclosed arms (transfer latency 1, TL 1) was recorded. If the mice failed to enter the enclosed arms within 90 s, they were placed at one of the enclosed arm with permission to explore the plus maze for additional 60 s. A criterion of an animal’s entry into the enclosed arm was crossing it with all four legs of an imaginary line separating the enclosed arm from the central space. A retention session followed 24 h after the acquisition session (on day 2). The mice were put into one of the open arms and the transfer latency 2 (TL 2) was recorded again. If the mice did not enter the enclosed arm within 90 s, the test was stopped and TL 2 was recorded as 90 s. TL 2 was utilized as an index of learning and memory processes. Prolongation of TL 2 shows that a drug has an amnesic effect, while the shortage of TL 2 means that the drug improves memory in mice relative to the control groups (Itoh et al. 1990; Hlinak and Krejci 2002; Orzelska et al. 2013). The plus maze was cleaned after each mouse to avoid the presence of olfactory trails.

### Novel Object Recognition Test (NOR)

An object recognition test was performed as described elsewhere (Ennaceur and Delacour 1988; Orzelska et al. 2015). An apparatus included a square open box, made of plexiglass (63 cm long × 44.5 cm high × 44 cm wide) and illuminated by a lamp (light intensity, 10 lx), suspended 50 cm above the box. Objects to be discriminated, made either of wood or plastic, were in two different shapes: block and ball. The objects were too heavy to be displaced by the animals. A day before the test, each rat was placed in the empty apparatus for 2 min to get used to the environment. On the experimental day, the animals were participating in two trials, spaced by a 1-h interval. The first trial (acquisition trial, T1) lasted 5 min and the second one (test trial, T2) was 3 min long. During T1, the apparatus contained two identical objects (wooden blocks), placed in two opposite corners, 10 cm from the sidewall. A rat was always placed in the middle of the box. After T1, the rat was put back into its home cage. Subsequently, after 1 h, T2 was performed. During T2, a new object (N) replaced one of the samples presented in T1; therefore, the rats were re-exposed to two objects: familiar (F) and N. In order to avoid the presence of olfactory trails, the apparatus and the objects were cleaned after each rat. Exploration was defined as follows: directing the nose toward the object at a distance of no more than 2 cm and/or touching the object with nose. Turning around or sitting on the object was not considered as exploratory behavior. Time periods, spent by rats in exploring each object during T1 and T2 tests, were recorded manually with a stopwatch. Discrimination between F and N during T2 was measured by comparing a time period, spent for exploration of the F object with time spent for exploration of the N object. Memory was evaluated with the discrimination index (DI), calculated for each animal by the following formula: (N − F)/(N + F), corresponding to the difference between exploration time periods for N and F, adjusted for the total exploration time period of both objects in T2. A higher DI is considered to reflect stronger memory retention for familiar objects elsewhere (Bertaina-Anglade et al. 2006; Orzelska et al. 2015).

### Locomotor Activity Test

Locomotor activity of individual rats was recorded, using a photocell device (plexiglass boxes—square cages, 60 cm on each side; Porfex, Bialystok, Poland) at a sound-attenuated experimental room, under moderate illumination (10 lx). Ambulatory activity (distance traveled) was measured by two rows of infrared light–sensitive photocells, installed along the long axis, 45 and 100 mm above the floor. Total horizontal activity (the distance traveled in meters) was recorded for a 15-min time period (Marszalek-Grabska et al. 2018).

The locomotor activity of individual mice was recorded using a photocell apparatus (round plexiglass cage, 32 cm in diameter, Multiserv, Lublin, Poland). The cages were equipped with one row of infrared light–sensitive photocells (2 emitters and 2 sensors) located 1 cm above the floor. Locomotor activity was recorded by the number of photocell interruptions of each mouse for a total period of 10 min (Vogel and Vogel 1997). The animals were placed individually into cages, 30 min after the injection of DZ or FNZ and 35 min after the injection of sildenafil or MB.

### Treatment

Sildenafil (1.25, 2.5, and 5 mg/kg, ip) (Devan et al. 2006; Tahsili-Fahadan et al. 2006) and MB (2.5, 5, and 10 mg/kg, ip) (Riha et al. 2005; Tahsili-Fahadan et al. 2006) were administered 35 min before T1, alone. In order to evaluate the influence of sildenafil or MB on DZ- or FNZ-treated rodents, sildenafil or MB were administered 5 min prior to DZ or FNZ injections. The route of (ip) administration of sildenafil and MB and the pretreatment time before testing of its effect were based upon information from previous experiments (Dhir and Kulkarni 2007; Talarek et al. 2010).

### Statistical Analysis

Based on TL2 data, DI values, the distance traveled or number of beam breaks for the co-administration of “sildenafil and DZ or sildenafil and FNZ,” and also of “MB and DZ or MB and FNZ,” were analyzed by two-way analysis of variance (ANOVA), with the drug treatment (saline and DZ or saline and FNZ) as factor 1 and the drug pretreatment (saline, sildenafil, or MB) as factor 2. In those cases that the interaction between treatment and pretreatment was significant or quite significant, Bonferroni’s post hoc test was applied. The index of probability of 0.05 or less (*p* < 0.05) was considered significant in comparative analysis. The data are presented as means ± standard errors of means (S.E.M.) at TL2 or DI values or distance segments, traveled in meters or number of beam breaks. Each animal group consisted of 10 animals. All figures were prepared using GraphPad Prism version 5.00 for Windows, GraphPad Software (San Diego, California, USA), www.graphpad.com.

## Results

### Effects of MB on DZ- or FNZ-Induced Memory Impairment in the mEPM Test

In the first trial (pre-test), no significant differences in TL1 values were identified between all groups (*p* > 0.05) (data not presented).

**Fig. 1:**
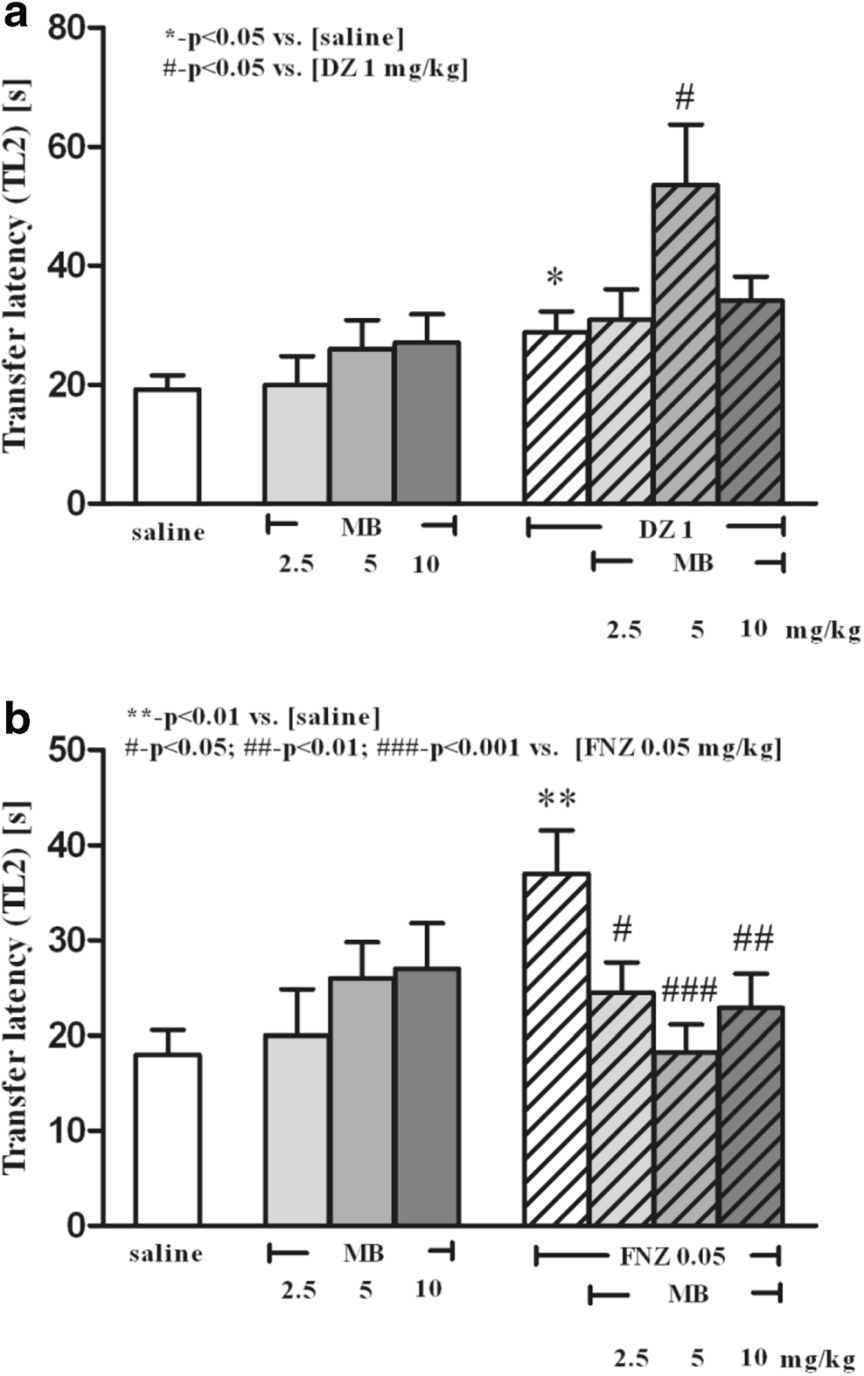
The influence of methylene blue (MB; 2.5, 5, and 10 mg/kg, ip) on the DZ-induced (1 mg/kg, sc) (**a**) and FNZ-induced (0.125 mg/kg, sc) (**b**) memory impairment of mice in the mEPM. MB was injected 5 min prior to administration of BZs, whereas BZs were injected 30 min prior to testing on the first trial. The data are expressed as mean ± SEM transfer latency on the second trial (TL2 in seconds). *p < 0.05, **p < 0.01 vs. saline control group; ^#^*p* < 0.05, ^##^*p* < 0.01, ^###^*p* < 0.001 vs. DZ-treated (**a**) or FNZ-treated (**b**) group (Bonferroni’s test)

There was a statistically significant effect caused by pretreatment with MB [*F*_(3,56)_ = 3,28; *p* = 0.0276] and DZ or saline treatment [*F*_(1,56)_ = 12,19; *p* = 0.0009]. The acute ip injection of MB (5 mg/kg), before DZ administration (1 mg/kg, sc), enhanced DZ-induced memory deficits, elongating the TL2 time period in the second trial, as compared with the DZ group (*p* < 0.05, post hoc Bonferroni’s test; Fig. 1a).

There was a statistically significant interaction between MB pretreatment and FNZ treatment [*F*_(3,53)_ = 3.02; *p* = 0.0379]. The acute ip injection of MB (2.5, 5, and 10 mg/kg) prevented FNZ-induced (0.125 mg/kg, sc) memory deficits (*p* < 0.05 for MB 2.5 mg/kg, *p* < 0.001 for MB 5 mg/kg, *p* < 0.01 for MB 10 mg/kg, post hoc Bonferroni’s test; Fig. 1b).

### Effects of Sildenafil on DZ- and FNZ-Induced Memory Impairment in the mEPM Test

In the first trial (pre-test), no significant differences in TL1 values were identified between all groups (*p* > 0.05) (data not presented).

**Fig. 2:**
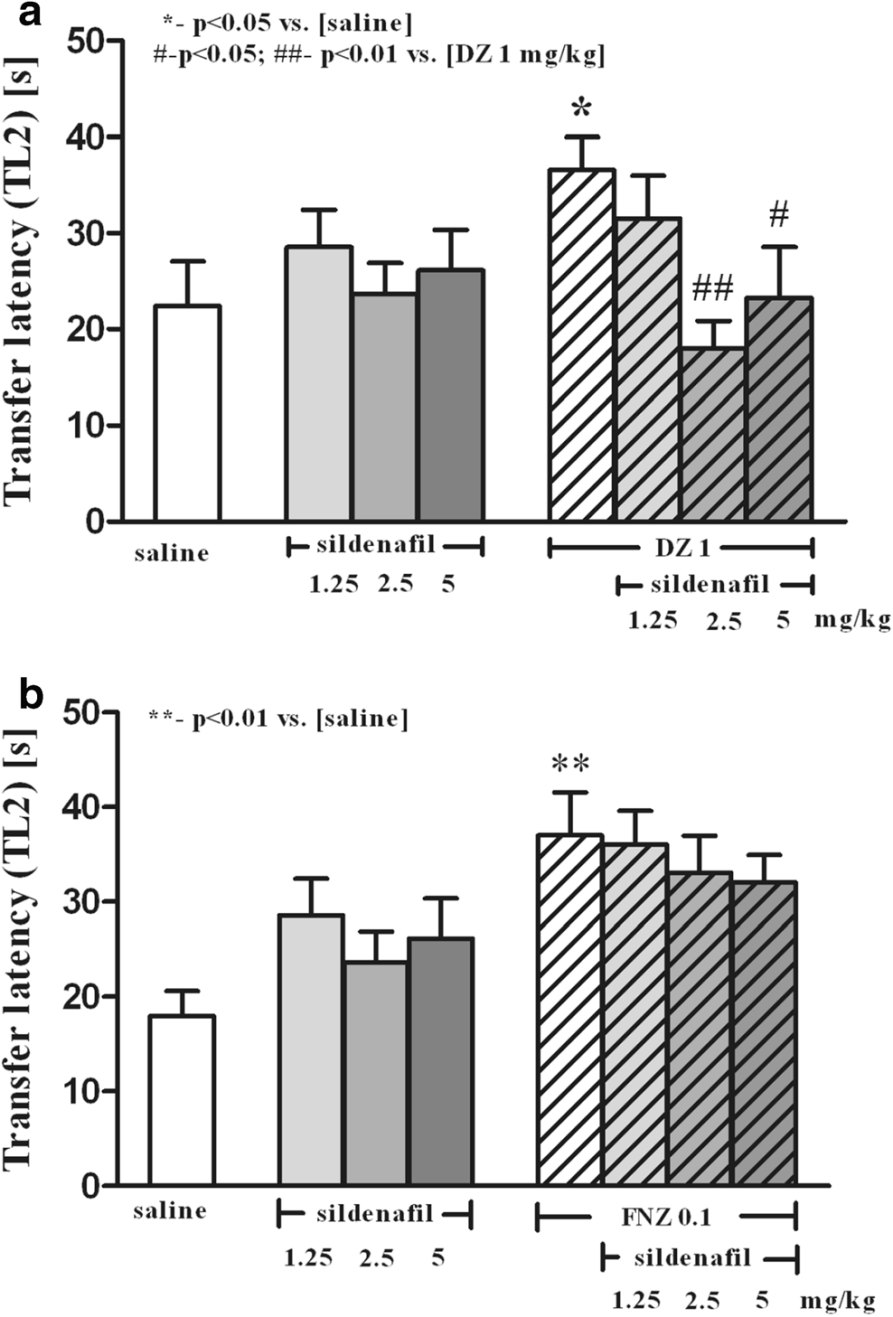
The influence of sildenafil (1.25, 2.5, 5, and 10 mg/kg, ip) on the DZ-induced (1 mg/kg, sc) (**a**) and FNZ-induced (0.1 mg/kg, sc) (**b**) memory impairment of mice in the mEPM. Sildenafil was injected 5 min prior to administration of BZs, whereas BZs were injected 30 min prior to testing on the first trial. The data are expressed as mean ± SEM transfer latency on the second trial (TL2 in seconds). *p < 0.05, **p < 0.01 vs. saline control group; ^#^*p* < 0.05, ^##^*p* < 0.01 vs. DZ-treated group (**a**) or FNZ-treated group (**b**) group (Bonferroni’s test)

There was a statistically significant effect caused by pretreatment with sildenafil [*F*_(3,54)_ = 2.94; *p* = 0.0413] and interaction between sildenafil pretreatment and DZ treatment [*F*_(3,54)_ = 3.15; *p* = 0.0323]. The acute ip injection of sildenafil (2.5 and 5 mg/kg) reduced DZ-induced (1 mg/kg, sc) memory deficits, shortening the TL2 time period in the second trial, as compared with the DZ group (*p* < 0.01 and *p* < 0.05, respectively, post hoc Bonferroni’s test; Fig. 2a).

There was a statistically significant treatment effect—FNZ 0.1 mg/kg [*F*_(1,56)_ = 11.4; *p* = 0.0013]. The acute ip injection of sildenafil (1.25, 2.5, and 5 mg/kg) did not change FNZ-induced (0.1 mg/kg, sc) memory deficits (Fig. 2b).

### Effects of Treatments on the Locomotor Activity of Mice

There was no significant difference between the groups, regarding the effects of either a single DZ (1 mg/kg), FNZ (0.05 and 0.1 mg/kg), MB (2.5, 5, and 10 mg/kg), or sildenafil (1.25, 2.5, and 5 mg/kg) injection on the total number of beam breaks (*p* > 0.05; Table 1 A–E and H). No significant differences in the total number of beam breaks values were identified between the groups receiving either DZ (1 mg/kg) or (FNZ 0.05 and 0.1 mg/kg) with MB (2.5, 5, and 10 mg/kg) or with sildenafil (1.25, 2.5, and 5 mg/kg) (*p* > 0.05; Table 1 F, G, I, and J).

**Table 1:** Effect of treatments on spontaneous locomotor activity in mice treated with MB or sildenafil and DZ or FNZ

	Treatment	Number of beam breaks (10 min)
A	Saline	159.5 ± 8.45
B	DZ 1 mg/kg	158.2 ± 13.55
C	FNZ 0.05 mg/kg	188 ± 17.87
D	FNZ 0.1 mg/kg	165.3 ± 14.44
E	MB 2.5 mg/kg	133.3 ± 12.11
	MB 5 mg/kg	144.9 ± 9.19
	MB 10 mg/kg	156.7 ± 13.4
F	DZ 1 mg/kg + MB 2.5 mg/kg	169.5 ± 19.5
	DZ 1 mg/kg + MB 5 mg/kg	184.4 ± 24.19
	DZ 1 mg/kg MB 10 mg/kg	152.8 ± 17.46
G	FNZ 0.05 mg/kg + MB 2.5 mg/kg	194.1 ± 14.87
	FNZ 0.05 mg/kg + MB 5 mg/kg	188.3 ± 16.96
	FNZ 0.05 mg/kg + MB 10 mg/kg	162.7 ± 21.6
H	Sildenafil 1.25 mg/kg	161.3 ± 15.6
	Sildenafil 2.5 mg/kg	174.1 ± 10.61
	Sildenafil 5 mg/kg	180.9 ± 18.36
I	DZ 1 mg/kg + sildenafil 1.25 mg/kg	157.9 ± 8.32
	DZ 1 mg/kg + sildenafil 2.5 mg/kg	179.5 ± 16.62
	DZ 1 mg/kg + sildenafil 5 mg/kg	175.9 ± 21.45
J	FNZ 0.1 mg/kg + sildenafil 1.25 mg/kg	174.1 ± 26.66
	FNZ 0.1 mg/kg + sildenafil 2.5 mg/kg	186.1 ± 9.73
	FNZ 0.1 mg/kg + sildenafil 5 mg/kg	168.6 ± 12.67

MB or sildenafil was injected 5 min prior to administration of BZ, whereas BZs were injected 30 min prior to the test. MB or sildenafil given alone was injected 35 min before the test. The data are expressed as mean ± S.E.M of the total number of beam breaks within 10 min

### Effects of MB on DZ- or FNZ-Treated Rats in the NOR Test

No difference was observed in any group during T1, when exploration time periods were compared for location of two identical objects in two opposite corners (the data not shown).

Two-way ANOVA revealed statistically significant effects of BZs or saline treatment [*F*_(2,55)_ = 5.87; *p* = 0.0049], pretreatment with MB [*F*_(3,55)_ = 3.14; *p* = 0.0323], and interaction between MB pretreatment and BZs treatment [*F*_(6,55)_ = 6.75; *p* < 0.0001].

**Fig. 3:**
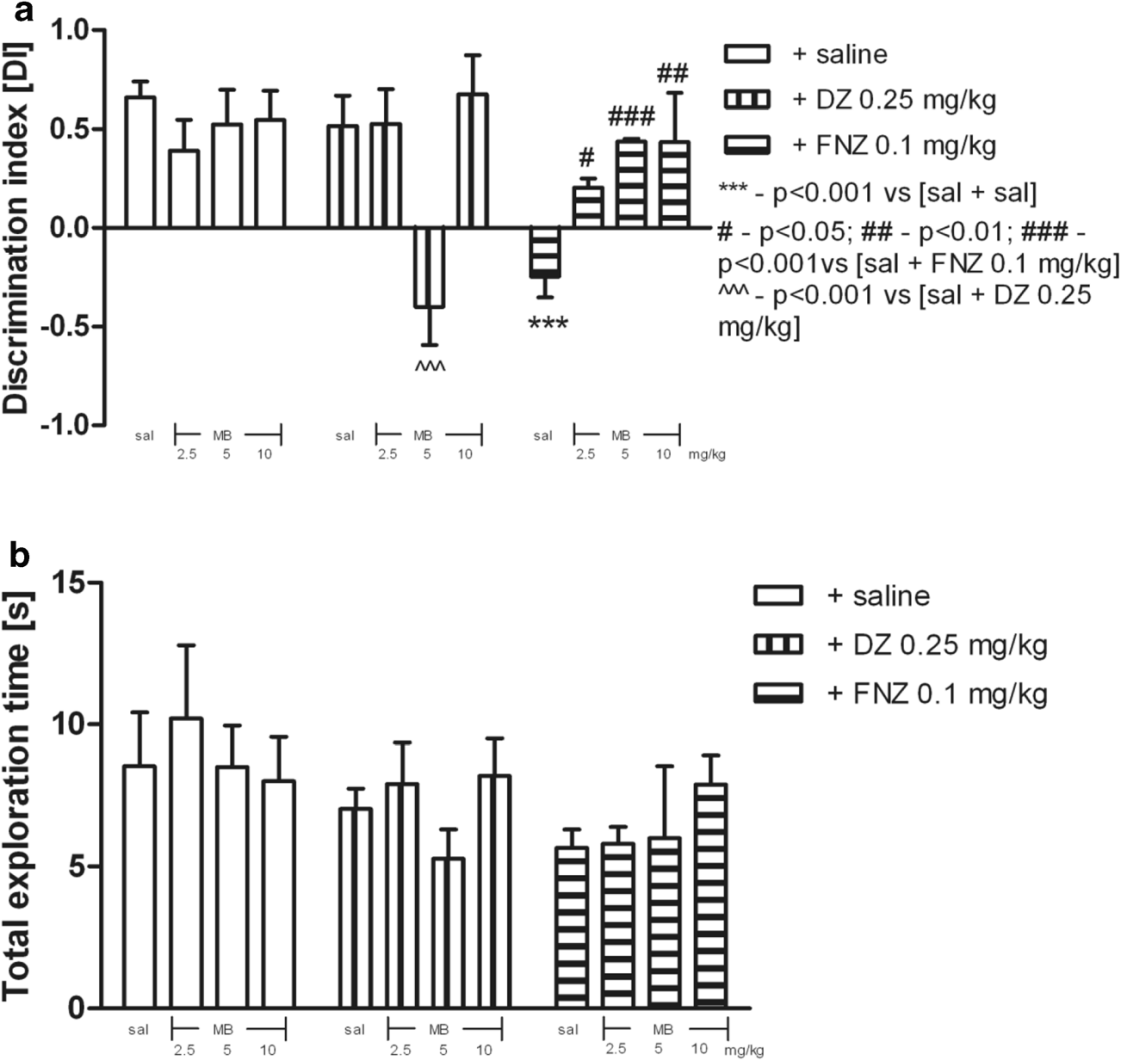
The influence of MB pretreatment (2.5, 5, and 10 mg/kg, ip) on rats performance in the object recognition task after DZ (0.25 mg/kg, sc) or FNZ (0.1 mg/kg, sc) treatment (**a**). Total exploration time displayed by different groups of rats in the object recognition task in T2 (**b**). MB was injected 5 min prior to administration of BZs, whereas BZs were injected 30 min prior to testing in the first trial. The data are expressed as mean ± SEM values. ***p < 0.001 vs saline control group; ^^^^^*p* < 0.001 vs DZ-treated group; ^#^*p* < 0.05, ^##^*p* < 0.01, and ^###^*p* < 0.001 vs FNZ-treated group (Bonferroni’s test)

The acute ip injection of MB (5 mg/kg) before DZ administration (0.25 mg/kg) induced memory deficits, as “MB-treated and DZ-treated” rats did not discriminate between novel and familiar objects during T2 test with respect to their counterparts on saline and DZ (0.25 mg/kg) (*p* < 0.001, post hoc Bonferroni’s test; see Fig. 3a).

The acute ip injection of MB (2.5, 5, and 10 mg/kg) before FNZ administration (0.1 mg/kg, sc) prevented FNZ-induced memory deficits, as the “MB-treated (2.5, 5, and 10 mg/kg) and FNZ-treated (0.1 mg/kg)” rats discriminated better the novel objects vs. the familiar objects during T2, when compared with their counterparts on saline and FNZ (0.1 mg/kg) (*p* < 0.05 for MB 2.5 mg/kg, *p* < 0.001 for MB 5 mg/kg, and *p* < 0.01 for MB 10 mg/kg, post hoc Bonferroni’s test; see Fig. 3a).

As shown in Fig. 3b in this set of experiments, total exploration time was unchanged.

### Effects of Sildenafil on DZ- or FNZ-Treated Rats in the NOR Test

No difference was observed between any groups during T1, when exploration time periods were compared for location of two identical objects in two opposite corners (the data not shown).

Two-way ANOVA revealed statistically significant effects of BZs or saline treatment [*F*_(2,73)_ = 14.39; *p* < 0.0001], pretreatment with sildenafil [*F*_(3,73)_ = 4.68; *p* = 0.048], and interaction between sildenafil pretreatment and BZs treatment [*F*_(6,73)_ = 7.66; *p* < 0.0001].

**Fig. 4:**
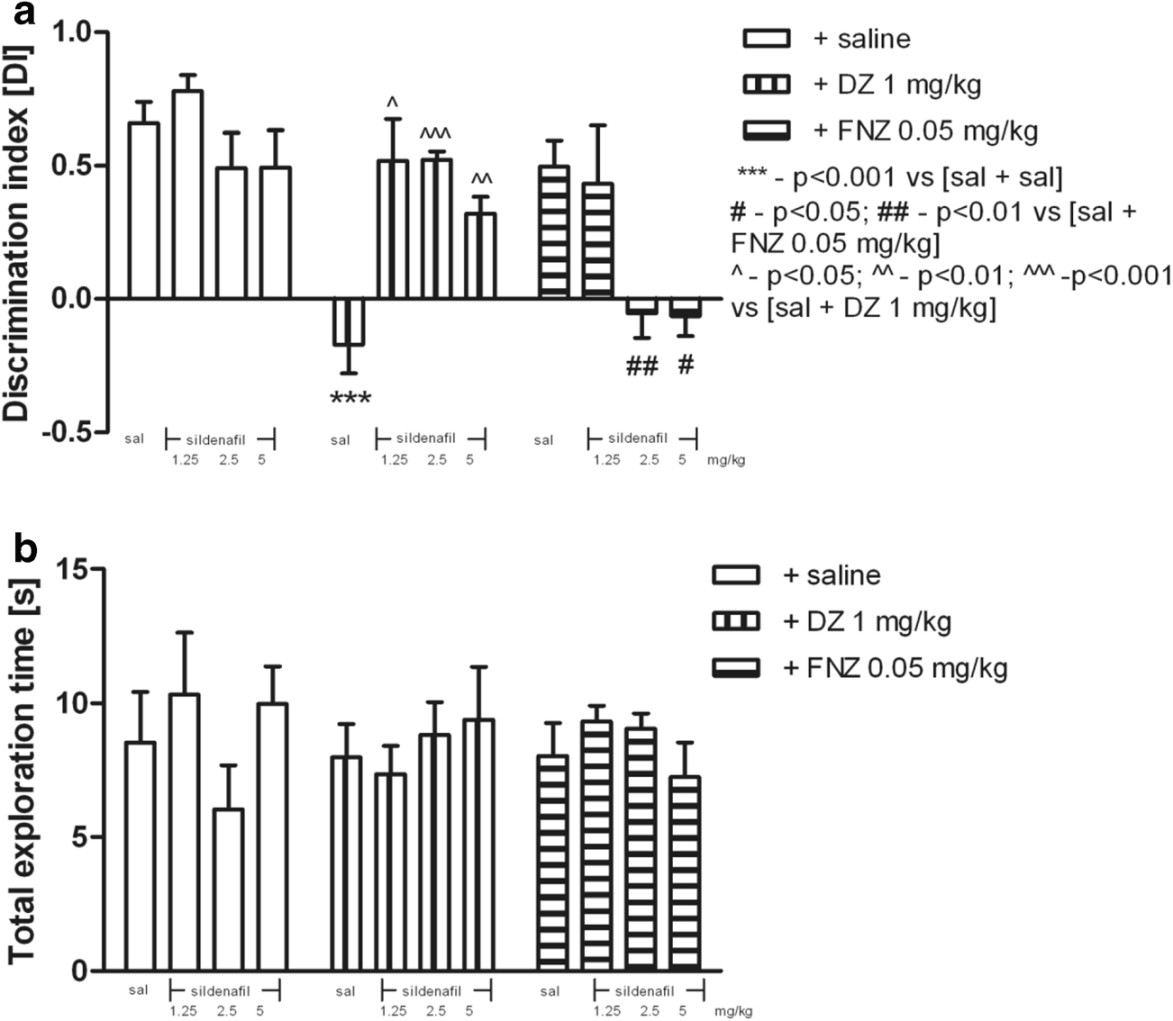
The influence of sildenafil pretreatment (1.25, 2.5, and 5 mg/kg, ip) on rats performance in the object recognition task after DZ (1 mg/kg, sc) or FNZ (0.05 mg/kg, sc) treatment (**a**). Total exploration time displayed by different groups of rats in the object recognition task in T2 (**b**). Sildenafil was injected 5 min prior to administration of BZs, whereas BZs were injected 30 min prior to testing in the first trial. The data are expressed as mean ± SEM values. ***p < 0.001 vs saline control group; ^^^*p* < 0.05, ^^^^*p* < 0.01, and ^^^^^*p* < 0.001 vs DZ-treated group; ^#^*p* < 0.05 and ^##^*p* < 0.01 vs FNZ-treated group (Bonferroni’s test)

The acute ip injection of sildenafil (1.25, 2.5, and 5 mg/kg) prevented DZ-induced (1 mg/kg, sc) memory deficits, as the “sildenafil-treated (1.25, 2.5, and 5 mg/kg) and DZ-treated (1 mg/kg)” rats discriminated much better the novel objects vs. the familiar objects during T2, when juxtaposed with their counterparts on saline and DZ (1 mg/kg) (*p* < 0.05 for sildenafil 1.25 mg/kg, *p* < 0.001 for sildenafil 2.5 mg/kg, and *p* < 0.01 for sildenafil 5 mg/kg, post hoc Bonferroni’s test; see Fig. 4a).

The acute ip injection of sildenafil (2.5 and 5 mg/kg) before FNZ (0.05 mg/kg) induced memory deficits, as “sildenafil-treated (2.5 and 5 mg/kg) and FNZ-treated (0.05 mg/kg)” rats did not discriminate between novel and familiar objects during T2 test with respect to their counterparts on saline and FNZ (0.05 mg/kg) (*p* < 0.01 for sildenafil 2.5 mg/kg and *p* < 0.05 for sildenafil 5 mg/kg, post hoc Bonferroni’s test; see Fig. 4a).

As shown in Fig. 4b in this set of experiments, total exploration time was unchanged.

### Effects of Treatments on the Locomotor Activity of Rats

There was no significant difference between the groups, regarding the effects of either a single DZ (0.25 or 1 mg/kg), FNZ (0.05 or 0.1 mg/kg), MB (2.5, 5, and 10 mg/kg), or sildenafil (1.25, 2.5, and 5 mg/kg) injection on the total distance traveled by rats (*p* > 0.05; Table 2 A–D and G). No significant differences in the total distance values were identified between the groups receiving either DZ (0.25 or 1 mg/kg) or FNZ (0.05 or 0.1 mg/kg) with MB (2.5, 5, and 10 mg/kg) or with sildenafil (1.25, 2.5, and 5 mg/kg) (*p* > 0.05; Table 2 E, F, H, and J).

**Table 2:** Effect of treatments on locomotor activity in rats treated with MB or sildenafil and DZ or FNZ

	Treatment	Mean of the distance traveled ± SEM [m] within 15 min
A	Saline	23.40 ± 2.63
B	DZ 1 mg/kg	27.97 ± 2.48
C	FNZ 0.05 mg/kg	25.25 ± 3.07
D	sildenafil 1.25 mg/kg	22.97 ± 1.84
	sildenafil 2.5 mg/kg	28.50 ± 1.54
	sildenafil 5 mg/kg	21.34 ± 6.12
E	DZ 1 mg/kg + sildenafil 1.25 mg/kg	27.97 ± 4.80
	DZ 1 mg/kg + sildenafil 2.5 mg/kg	19.43 ± 4.07
	DZ 1 mg/kg + sildenafil 5 mg/kg	18.92 ± 1.59
F	FNZ 0.05 + sildenafil 1.25 mg/kg	24.06 ± 3.84
	FNZ 0.05 + sildenafil 2.5 mg/kg	27.83 ± 1.90
	FNZ 0.05 + sildenafil 5 mg/kg	26.26 ± 3.58
G	MB 2.5 mg/kg	23.24 ± 1.74
	MB 5 mg/kg	25.50 ± 1.82
	MB 10 mg/kg	20.66 ± 5.16
H	DZ 1 mg/kg + MB 2.5 mg/kg	19.87 ± 3.75
	DZ 1 mg/kg + MB 5 mg/kg	20.45 ± 3.07
	DZ 1 mg/kg + MB 10 mg/kg	19.92 ± 2.96
I	FNZ 0.05 + MB 2.5 mg/kg	22.26 ± 3.33
	FNZ 0.05 + MB 5 mg/kg	24.56 ± 2.15
	FNZ 0.05 + MB 10 mg/kg	22.05 ± 1.89

MB or sildenafil was injected ip 5 min prior to administration of BZ, whereas BZs were injected sc 30 min prior to the test. The data are expressed as mean ± SEM of total distance traveled in meters within 15 min

## Discussion

Scientists suggested a dichotomy in the temporal lobe and in the prefrontal structures, mediating object and spatial memory. It is, therefore, plausible that recognition memory and spatial memory processes activate different parts of rat brain (Steckler et al. 1998). Thus, in the present study, we used two different models of memory—NOR and mEPM. NOR test is a behavioral test that constitutes one of the animal models of human amnesia. It uses innate tendency of rodents to examine novel objects (snoopy nature of rodents) as well as it evaluates their ability of recognition memory. This behavior is being measured by the differences in time required to explore novel and familiar objects. One of the advantages of NOR test is the short time required for a whole experiment. Moreover, it does not require external stimulus to motivate animals (Antunes and Biala 2012). Another animal model of amnesia is mEPM, which defines spatial memory in rodents. It uses natural tendency of them to avoid open and elevated spaces. Learning and memory processes are related to time changes which rodent needs to move from an open to a close area (Orzelska et al. 2013; Yildiz Akar et al. 2007).

The presented experiments confirmed memory impairments after administration of DZ (1 mg/kg) or FNZ (0.05 and 0.1 mg/kg) in mice in the mEPM test. TL 2 in the retention sessions have been longer compared with the TL 1 in the pre-test session. The NOR test conducted in rats proved that either DZ (1 mg/kg) or FNZ (0.1 mg/kg) induced anterograde amnesia observed through decreased DI values. These results are consistent with known spatial and recognition memory impairments induced by BZs in rodents (Bertaina-Anglade et al. 2006; Prabhakar et al. 2008).

In the present study, there is an interesting dosage-related effect we noticed in the NOR test. FNZ given at a dose of 0.05 mg/kg was not able to affect DI. FNZ at higher dose (0.1 mg/kg) induced memory impairment. A similar observation was recorded with DZ. When DZ was given at a dose of 0.25 mg/kg, no changes were observed in the DI values. In contrast, DZ administered at a dose of 1 mg/kg significantly decreased the DI. This observation is consistent in general dose-related responses among BZs. BZs dose-response relationship has a linear slope to some point and then it can be observed a deviation from a linear dose-dependent state. BZs belong to a group of drugs with flatter curves which are safer for clinical uses (Katzung et al. 2019).

In order to rule out possibility of sedative effects of the BZs on animal behavior in mEPM and NOR tests, we performed the locomotor activity test. DZ and FNZ given at doses 0.25 and 1 mg/kg and 0.05 and 0.1 mg/kg appropriately did not have an influence on the locomotor activity of mice and rats. Thus, the presented memory impairment induced by BZs in both tests did not result from the sedative actions of DZ and FNZ.

A growing body of evidence shows that NO-dependent upregulation of sGC is involved in synaptic plasticity and enhances memory formation (Pitsikas 2015). To our knowledge, no previous studies have reported the involvement of the downstream NO:cGMP signaling pathway in the BZs-induced memory disturbances. In this study, we demonstrated that sildenafil (PDE-5 inhibitor) and MB (sGC inhibitor) affect memory impairment induced by two BZs—DZ and FNZ—in two behavioral tests.

MB administered at a dose of 5 mg/kg potentiated memory impairment evoked by DZ in the mEPM test and induced amnesic effect of DZ in the NOR test. In the case of memory impairment induced by FNZ, the effect of MB in the mEPM test was opposite—MB reversed FNZ-induced memory impairment. In the NOR test, we observed the same effect as in the mEPM. Rats treated with FNZ (0.1 mg/kg) and MB at all tested doses (2.5, 5, and 10 mg/kg) were capable of discriminating between familiar and new objects 1 h after the pre-test. MB improved significantly their object recognition abilities. Our results on the interaction between MB and DZ in two memory tests are consistent with previous results showing that the inhibition of the NO:cGMP signaling pathway by NOS inhibitors or sGC inhibitors leads to the impairment of memory processes (Pitsikas 2015; Shen et al. 2012). It was proven that 1H-[1,2,4] oxadiazolo-[4,3-a]quinoxalin-1-one (ODQ), another sGC inhibitor, blocked morphine-induced reward memory assessed in conditioned place preference in rats (Shen et al. 2012). On the other hand, Deiana et al. (2009) demonstrated that MB administered ip reversed in a dose-dependent manner the amnesic effect of scopolamine in the water maze test in mice. The ambiguous effects of MB on memory processes observed in our study are difficult to explain. As it was demonstrated in recent studies, the mechanism of MB action is complex and not fully determined. MB, in addition to the cGMP level regulation, is also recognized as a significant antioxidant. Acting as a cytochrome *c* oxidase activator, the compound improves cellular metabolism. The beneficial effect of MB on cognitive processes by improving cellular metabolism was described in another study (Oz et al. 2011). Bearing it in mind, we cannot exclude that inhibition of the amnesic effect of FNZ by MB observed in the presented study is related to the antioxidant action of MB.

Sildenafil is a medication used to treat erectile dysfunction and pulmonary arterial hypertension. Sildenafil acts by blocking PDE5, an enzyme that promotes breakdown of cGMP (Orzelska and Talarek 2008). The presented experiments demonstrated that sildenafil (2.5 and 5 mg/kg) reduced spatial memory impairments imposed by DZ (1 mg/kg) in the mEPM test as well as new object recognition after administration of the same dose of DZ in the NOR test. In the NOR test, sildenafil in two doses (2.5 and 5 mg/kg) decreased DI values among rats treated with ineffective dose of FNZ (0.05 mg/kg) but sildenafil did not exert this effect when administered at a dose of 1.25 mg/kg.

It is known that PDE5 inhibitors activate the NO:cGMP pathway (Reneerkens et al. 2012; Tahsili-Fahadan et al. 2006). In comparison with NO donors, they lack of direct action on NO production (Gholipour et al. 2009). It has been documented that PDE5 inhibitors prolong and enhance the response of the target cell to NO (Puzzo et al. 2008; Reneerkens et al. 2012). Many recent behavioral studies indicate that increased cGMP level, by inhibiting PDE, and especially PDE5, has a positive effect on learning and memory processes (Devan et al. 2006; Prickaerts et al. 2005; Reneerkens et al. 2012). Devan et al. (2006) have suggested that sildenafil may serve as a cognitive enhancer by modulating central NO:cGMP signal transduction. They showed that sildenafil reversed a learning impairment in rats induced by systemic inhibition of NOS by N^G^-nitro-l-arginine methyl ester (l-NAME). In other studies, it was reported that sildenafil improved the process of acquisition and consolidation of object information in rats (Prickaerts et al. 2005). Reneerkens et al. (2012) indicated that vardenafil (the other PDE5 inhibitor) improved object recognition memory where memory was disrupted by the muscarinic antagonist scopolamine or the NMDA antagonist MK-801. In addition, PDE5 was found in the hippocampus, cortex, and cerebellum—structures involved in memory formation—in rodents and humans, which may also indicate the participation of PDE5 in the mechanisms of cognitive processes (Puzzo et al. 2008; Reneerkens et al. 2012). Our results confirm and extend previous reports of the ability of PDE inhibitors to affect cognitive and memory processes in rodents.

The present study revealed differences in the effects of NO modulators on the action of DZ and FNZ on learning and memory processes in rodents. These findings seem to be consistent with data reported in our previous studies (Orzelska et al. 2013, 2015; Orzelska-Gorka et al. 2016). Our earlier studies demonstrated that l-NAME, a non-selective NOS inhibitor, and 7-nitroindazole (7-NI), a selective inhibitor of nNOS, enhanced DZ-induced, but prevented FNZ-induced memory impairment (acquisition process) in the mEPM (Orzelska et al. 2013) and NOR (Orzelska et al. 2015; Orzelska-Gorka et al. 2016) tests.

It is very interesting to observe a different orientation of the interactions between the modification of NO-cGMP pathway and actions of two BZs which belongs to the same therapeutic group. But it should be underlined that a chemical structure of those BZs is different especially when it comes to substituent in position 7. DZ is substituted for Cl in the position 7, whereas FNZ binds with a nitro group. The nitro group is reported to induce hypnotic activity (Ben-Cherif et al. 2010) but as well can help with a faster passage of the blood-brain barrier due to it is non-polar feature. The last one is responsible for very fast onset of actions, which was a reason to use FNZ commonly for anesthesia. This quick onset of action is also a reason why FNZ has been withdrawn from treatment in several countries due to its usage as a day-rape substance. But it should be considered that the above consideration is rather speculative than based on evidence. The potential relationship between chemical structure of BZs and the obtained results is worthy of further investigation.

Another explanation that may be provided for divergent action of MB or sildenafil on the effects of DZ and FNZ is their selective actions at specific GABA_A_ receptor subunits. To explore this hypothesis, experiments with the use of point-mutated animals would be needed to determine which GABA_A_ units are responsible for particular mechanism of action of DZ and FNZ.

The results of behavioral studies investigating interactions between BZs and NO in animals have been not consistent. 7-NI was shown to block the anticonvulsant effect of DZ in picrotoxin-induced convulsions in rats (Paul and Ekambaram 2003). Another NOS inhibitor, N^G^-nitro-l-arginine (l-NOARG), a non-selective inhibitor of NOS, at a dose known to decrease level of NO, blocked the anxiolytic effect of chlordiazepoxide (CDZ) in mice (Elfline et al. 2004). Thus, the reduction of the NO level inhibited the effects of BZs. On the contrary, there are data indicating that the reduction of the NO level can enhance the action of BZs. Talarek and Fidecka (2003) and Deutsch et al. (1995) found that l-NAME, 7-NI, and MB potentiated the anticonvulsant effect of DZ and flurazepam in the pentylenetetrazole-induced (PTZ) and electroshock-induced seizure models in mice. l-NAME, 7-NI, and MB increased the hypnotic and analgesic effect of DZ, CDZ, and clonazepam in mice (Talarek and Fidecka 2002, 2004).

A pharmacologically induced increase of the endogenous NO level may also differently affect the action of BZs. For example, sildenafil enhanced the anticonvulsant effect of DZ in a mouse model of clonic seizures induced by PTZ (Gholipour et al. 2009). In turns, molsidomine, a donor of NO, remained without effect upon the protective activity of clonazepam in PTZ-induced clonic convulsions in mice (Tutka et al. 2002).

There is evidence of a significant contribution of NO in the development of tolerance to the coordination disturbance and sedative effects of DZ. Administration of compounds that increase the NO level, i.e., l-arginine, a substrate for NO synthesis or sildenafil, enhanced the development of tolerance and the use of inhibitors suppressed this phenomenon (Talarek et al. 2008, 2010).

It is noteworthy that in the present study, both compounds, sildenafil (1.25, 2.5, and 5 mg/kg) and MB (2.5, 5, and 10 mg/kg) when given alone, had no impact on the mEPM and NOR behavior. Even more, our results rule out the possibility that the interactions between NO-related compounds with BZs in NOR task might be caused by motivational factors. Total exploration times displayed by all groups during the choice trail (T2) were unchanged.

Based on obtained data, we can conclude that mechanism(s) related to NO may be involved into BZs-induced memory impairment in rodents. However, it is difficult to make a clear conclusion on the involvement of NO in this action of BZs and further studies are warranted.
